# Presence of Circulating miR-145, miR-155, and miR-382 in Exosomes Isolated from Serum of Breast Cancer Patients and Healthy Donors

**DOI:** 10.1155/2019/6852917

**Published:** 2019-02-12

**Authors:** Vianey Gonzalez-Villasana, Mohammed H. Rashed, Yessica Gonzalez-Cantú, Recep Bayraktar, Jorge Luis Menchaca-Arredondo, Jose Manuel Vazquez-Guillen, Cristina Rodriguez-Padilla, Gabriel Lopez-Berestein, Diana Resendez-Perez

**Affiliations:** ^1^Universidad Autónoma de Nuevo León, College of Biological Sciences, Department of Cellular Biology and Genetics, San Nicolás de los Garza, Nuevo León, Mexico; ^2^Department of Experimental Therapeutics, The University of Texas MD Anderson Cancer Center, Houston, TX, USA; ^3^Department of Pharmacology and Toxicology, Faculty of Pharmacy, Al-Azhar University, Cairo, Egypt; ^4^Universidad Autónoma de Nuevo León, College of Biological Sciences, Laboratory of Immunology and Virology, San Nicolás de los Garza, Nuevo León, Mexico; ^5^Universidad Autónoma de Nuevo León, College of Physics and Mathematics, Research Center in Physics and Mathematic Sciences, San Nicolás de los Garza, Nuevo León, Mexico; ^6^Department of Gynecologic Oncology, The University of Texas MD Anderson Cancer Center, Houston, TX, USA; ^7^Department of Center for RNAi and Non-coding RNA, The University of Texas MD Anderson Cancer Center, Houston, TX, USA

## Abstract

miR-145, miR-155, and miR-382 have been proposed as noninvasive biomarkers to distinguish breast cancer patients from healthy individuals. However, it is unknown if these three miRNAs are secreted by exosomes. Thus, we hypothesized that miR-145, miR-155, and miR-382 in breast cancer patients are present in exosomes. We isolated exosomes from serum of breast cancer patients and healthy donors, then we characterized them according to their shape, size, and exosome markers by scanning electron microscopy, atomic force microscopy, nanoparticle tracking analysis (NTA), and Western blot and determined the exosome concentration in all samples by NTA. Later, exosomal small RNA extraction was done to determine the expression levels of miR-145, miR-155, and miR-382 by qRT-PCR. We observed a round shape of exosomes with a mean size of 119.84 nm in breast cancer patients and 115.4 nm in healthy donors. All exosomes present the proteins CD63, Alix, Tsg, CD9, and CD81 commonly used as markers. Moreover, we found a significantly high concentration of exosomes in breast cancer patients with stages I, III, and IV compared to healthy donors. We detected miR-145, miR-155, and miR-382 in the exosomes isolated from serum of breast cancer patients and healthy donors. Our results show that the exosomes isolated from the serum of breast cancer patients and healthy donors contains miR-145, miR-155, and miR-382 but not in a selective manner in breast cancer patients. Moreover, our data support the association between exosome concentration and the presence of breast cancer, opening the possibility to study how miRNAs packaged into exosomes play a role in BC progression.

## 1. Introduction

Breast cancer (BC) is the most common cancer among women and the second cause of cancer deaths in women after lung cancer [1]. The overall five-year relative survival rate for women diagnosed with localized BC is 99%, whereas that for patients with distant-stage disease is 27% [2]. For these reasons, many efforts have been made to identify circulating noninvasive biomarkers for the diagnosis and prognosis of BC.

MicroRNAs (miRNAs) are noncoding RNAs of about 22 nucleotides in length, which regulate gene expression at the posttranscriptional level by binding to the 3′ untranslated region (UTR) of its target mRNA, leading to translational inhibition or mRNA degradation [3, 4]. In cancer, miRNA expression is dysregulated, showing a miRNA signature that has been associated with diagnosis and prognosis in cancer [5, 6]. miRNAs are present and highly stable in biofluids like urine, plasma, and serum [7–9]. These characteristics have allowed miRNAs to emerge as biomarkers in many types of cancer including BC [10, 11]. The miRNAs are carried out and protected from endogenous RNase activity in blood by their association with exosomes, argonaute-2 (Ago-2), and high-density lipoproteins [8, 12].

Exosomes are extracellular vesicles with a maximum size of 150 nm that can be found in urine, serum, plasma, breast milk, saliva, and other bodily fluids [13–15]. They are secreted by all types of cells and carry DNA, RNA, miRNAs, and proteins to recipient cells [12, 13]. Recently, exosomal miRNAs have gained attention, because of their potential activity as biomarkers in several types of cancer such as BC. Some of the exosome miRNAs proposed as diagnostic biomarkers in BC have been miR-1246, miR-21, miR-373, miR-182, miR-105, and miR-223-3p [16, 17]. For prognosis biomarkers, miR-340-5p, miR-17-5p, miR-130a-3p, and miR-93-5p have been suggested [18, 19].

Previously, we analyzed the expression levels of seven miRNAs (miR-10b, miR-21, miR-125b, miR-145, miR-155, miR-191, and miR-382) in the serum of Mexican patients with BC, and we found that miR-145, miR-155, and miR-382 potentially could be used as noninvasive biomarkers to distinguish BC patients from healthy controls [20]. miR-145 presents a tumor suppressor activity in BC, inhibiting cell proliferation, migration, and invasion [21–23]. miR-155 is involved in breast cancer progression, suppressing the function of tumor suppressors like TP53 [24] and also plays a role as tumor suppressor in BC [25]. miR-382 is reported to promote cell viability, migration, invasion, and survival in BC [26]. Although the function of these miRNAs was described, it is unknown whether these three miRNAs are secreted by exosomes in Mexican BC patients.

In this study, we proposed to determine whether serum-circulating miR-145, miR-155, and miR-382 are present in exosomes and whether there are differences in the concentration of exosomes between BC patients and healthy donors.

## 2. Materials and Methods

### 2.1. Patient Samples

Blood samples from Mexican patients with a confirmed diagnosis of ductal carcinoma (*n* = 20; 5 patients per breast cancer stage I-IV) and healthy donors (*n* = 5) were collected at the Center of Immunological Specialties and Cancer Treatment in San Nicolás de los Garza Nuevo León, México. The mean age of BC patients was 54.3 years and 52 for healthy donors. Most BC patients received therapy before the collection of blood samples. The comorbidities for healthy donors were diabetes, allergies, and hypercholesterolemia, and those for BC patients were hypertension, ankylosing spondylitis, osteoporosis, and hypothyroidism (Table 1). This study was approved by the Institutional Review Board of the College of Biological Sciences of the Universidad Autónoma de Nuevo León, according to Helsinki Declaration. Signed informed consent from all participants to be part in this study was obtained.

### 2.2. Serum Exosome Isolation

Blood from BC patients and healthy donors was obtained from February 2015 to May 2015. Serum was extracted by centrifugation (3000 rpm for 10 minutes at room temperature) immediately after collection and stored at -80°C in RNAse-free Eppendorf ™ tubes until use. Exosome isolation from serum samples were carried after two months of freezing, using ExoQuick Exosome Precipitation Solution (System Biosciences, Palo Alto, CA) following the manufacturer's protocol. Briefly, serum samples were thawed and centrifuged at 3000 x*g* for 15 minutes to discard cell debris. Next, we added in Eppendorf tubes 250 *μ*l of serum and 63 *μ*l of ExoQuick reagent. After 12 hours of incubation at 4°C, precipitated exosomes were collected by centrifugation at 1500 x*g* for 30 minutes.

### 2.3. Scanning Electron Microscopy (SEM)

We resuspended the exosome pellet extracted from the serum of BC patients in 300 *μ*l of 1X PBS, then we made a dilution of 1 : 100 in 1X PBS and a drop of 50 *μ*l was deposited in mica surfaces and dried overnight. Scanning electron micrographs of exosomes were obtained in SEM Nova Nano SEM 200 (FEI Company, Hillsboro, USA) using a low-vacuum (LV) detector.

### 2.4. Atomic Force Microscopy (AFM)

The exosomes isolated from the serum of BC patients were observed using an NT-MDT NTEGRA Prima AFM at room temperature, with a RTESPA probe (Bruker) of spring constant *k* = 40 N/m in intermittent contact mode. Images of height, deflection, and phase were obtained; images of 20 × 20, 10 × 10, and 5 × 5 *μ*m^2^ were captured systematically for each sample in at least three different regions. They were analyzed with WSXM software to observe the morphological aspect of exosomes [27].

### 2.5. Nanoparticle Tracking Analysis (NTA)

Exosome pellets were resuspended in 1 ml of 1X PBS and then diluted (1 : 50) to analyze the exosome size (nm) and concentration (particles/ml) in all samples using a NanoSight NS300 instrument, according to the manufacturer's instructions. The nanoparticle tracking analysis 3.2 software identifies and tracks individual vesicles that move under Brownian motion, which are recorded in a real-time video. The video is then analyzed by the NTA 3.2 software to calculate exosome size and concentration. All measurements were done by triplicate.

### 2.6. Western Blotting

Exosomes were lysed with ice-cold 1X RIPA buffer (Thermo Scientific, Pittsburgh, PA) and centrifuged at 13,000 rpm for 10 minutes at 4°C; supernatants were collected, and protein concentration was determined using the BCA Protein Assay (Thermo Scientific). Exosome lysates were prepared for loading in Laemmli loading buffer (Bio-Rad Laboratories, Hercules, CA) and electrophoresed using 4-15% gradient polyacrylamide gels (Bio-Rad). Next, proteins were transferred to nitrocellulose membranes (Bio-Rad). Membranes were blocked with 5% milk/TBS-T, rinsed with TBS-T buffer, and incubated with primary antibodies against the most common exosomal proteins CD63 (System Biosciences), Alix (Santa Cruz Biotechnology Inc., Santa Cruz, CA), Tsg (Santa Cruz Biotechnology), CD9, and CD81 (System Biosciences). After overnight incubation at 4°C, membranes were washed and incubated with a secondary antibody conjugated with horseradish peroxidase (HRP). An enhanced chemoluminescence detection kit (GE Healthcare, Piscataway, NJ) was used to visualize protein bands.

### 2.7. Exosomal RNA Isolation

The miRCURY™ RNA isolation kit for biofluids (Exiqon, Vedbaek, Denmark) was used for RNA extraction. Briefly, exosomes were resuspended in 200 *μ*l of RNase-free water, then 60 *μ*l of lysis solution BF was added. After 3 minutes of incubation at room temperature, the protein precipitation solution BF was added. Samples were vortexed, incubated for 1 minute at room temperature, and centrifuged at 11,000 x*g* for 3 minutes. Next, isopropanol was added, and samples were loaded onto a column followed by 3 washes and the RNA elution in 50 *μ*l of RNase-free water. The quality of the RNA isolated was determined by an Agilent 2100 Bioanalyzer (Agilent Technologies, Santa Clara, CA) with the RNA 6000 Pico Kit (Agilent Technologies) according to the manufacturer's protocol.

### 2.8. miRNA Detection by qRT-PCR

For the analysis of the expression of miR-145, miR-155, and miR-382 in exosomes isolated from BC patients and healthy donors, 150 ng of total RNA was reverse-transcribed to cDNA using the qScript microRNA cDNA Synthesis Kit (Quanta BioSciences, Gaithersburg, MD). Briefly, all reagents for the poly(A) tailing reaction were mixed and incubated for 60 minutes at 37°C followed by 5 minutes at 70°C. For the first-strand cDNA synthesis reaction, the incubation conditions were 20 minutes at 42°C followed by 5 minutes at 85°C. The qRT-PCR was performed by using PerfeCTa SYBR Green SuperMix (Quanta BioSciences) under the following conditions: 95°C for 2 minutes, 40 cycles of 95°C for 5 seconds, 60°C for 15 seconds, and 70°C for 15 seconds. All reactions were done by triplicate. The expression levels of miR-145, miR-155, and miR-382 were normalized using miR-16 as an endogenous reference gene (Supplementary Table 1 and Supplementary Figure 1). We also tested the expression of cel-miR-39 from *Caenorhabditis elegans* as an exogenous reference gene, which was added to the samples after the lysis step during exosomal RNA isolation and before the qRT-PCR (Supplementary Table 2 and Supplementary Figure 2). The relative expression of each miRNA was calculated using the 2^-ΔΔCt^ method [28]. miRNA primers were purchased from Quanta BioSciences.

### 2.9. Statistical Analysis

The statistical analyses were conducted using the GraphPad Prism software (GraphPad Software, San Diego, CA). Shapiro-Wilk and Kolmogorov-Smirnov tests were applied to determine if the data follows a normal distribution. The Student *t-*test was performed for data that was normally distributed. For data with nonparametric distribution, the Kruskal-Wallis test was used followed by the Mann-Whitney *U* test. For all assays *P* < 0.05 was considered statistically significant.

## 3. Results

### 3.1. Characterization of Serum Exosomes by SEM and AFM

**Figure 1:**
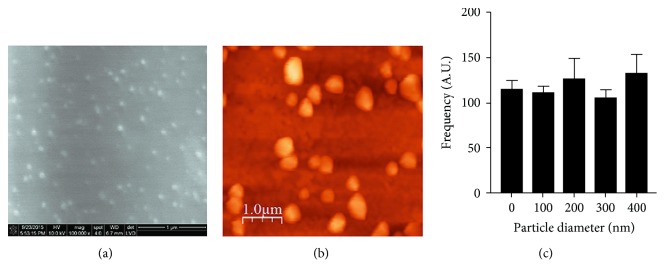
Characterization of exosomes by scanning electron microscopy (SEM) and atomic force microscopy (AFM). Representative (a) SEM and (b) AFM micrographs showing the round shape of exosomes isolated from serum of BC patients using ExoQuick Exosome Precipitation Solution (System Biosciences, Palo Alto, CA), scale bar = 1 *μ*m. (c) Histogram of the size distribution of exosomes from serum of BC patients, based on AFM data. The mean size of exosomes shown in the bell curve is 134.34 ± 4.45. Frequency is in arbitrary units (A.U.).

To determine whether miR-145, miR-155, and miR-382 are secreted by exosomes in breast cancer patients, we first isolated exosomes from serum of BC patients and healthy donors using ExoQuick reagent. Next, we performed the characterization of exosomes through their shape and size using SEM and AFM which showed a round shape of exosomal vesicles (Figures 1(a)–1(b)) and a mean size of 134.34 nm in exosomes isolated from BC patients (Figure 1(c)).

### 3.2. Serum Exosome Characterization by NTA and Western Blot

**Figure 2:**
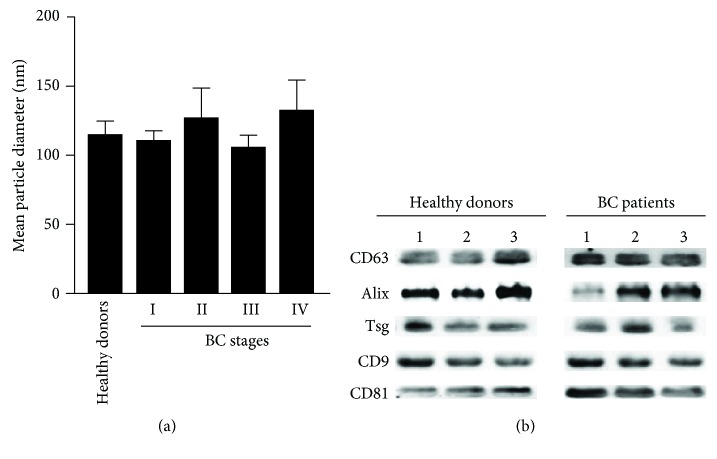
Characterization of exosomes by tracking analysis (NTA) and Western blot. (a) The size of the exosomes (nm) isolated from the serum of BC patients and healthy donor was determined through NTA using a NanoSight NS300 instrument (NanoSight Ltd., Amesbury, UK). All measurements were done by triplicate, and values are presented as mean ± SD. Statistical analysis was conducted by the Student *t-*test. ^∗^ *P* < 0.05; ^∗∗^ *P* < 0.001; *n* = 25 [5 healthy donors, 5 BC patients per BC stage (I-IV)]. (b) Immunobloting of the exosomal markers CD63, Alix, Tsg, CD9, and CD81. A concentration of 30 *μ*g of proteins was used. Enhanced chemoluminescence detection kit (GE Healthcare, Piscataway, NJ) was used to visualize protein bands. Three representative samples from healthy donors and BC patients are shown.

Exosome size was also determined by NTA, which showed a mean size of 119.84 nm in exosomes isolated from BC patients and 115.4 nm in those isolated from healthy donors. Moreover, we found that, compared with the size of exosomes isolated from serum of healthy donors (115.4 nm), the mean particle diameter in exosomes obtained from BC patients in stages I (111.68 nm), II (127.44 nm), III (106.76 nm), and IV (133.48 nm) was not significantly different (Figure 2(a)). The mean size found for BC exosomes was similar in both AFM and NTA analyses.

Next, we evaluated the presence of the exosomal markers CD63, Alix, Tsg, CD9, and CD81 in the vesicles isolated from the serum of BC patients and healthy donors and found them expressed in all of our samples (Figure 2(b)). Taken together, our observations suggest that the vesicles isolated from the serum of BC patients and healthy donors correspond to exosomes.

### 3.3. High Exosome Concentration in the Serum of BC Patients

**Figure 3:**
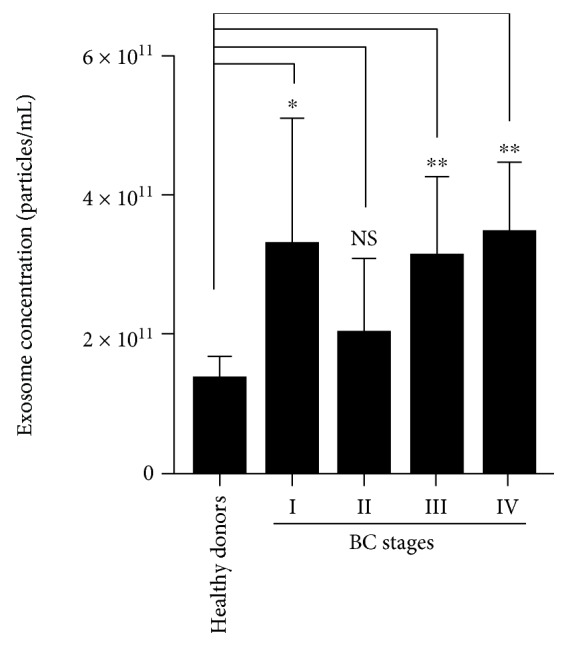
Concentration of exosomes from the serum of BC patients. Exosome concentration (particles/ml) was determined through NTA using a NanoSight NS300 instrument (NanoSight Ltd., Amesbury, UK). All measurements were done by triplicate, and values are presented as mean ± SD. Statistical analysis was conducted by the Student *t-*test. ^∗^ *P* < 0.05; ^∗∗^ *P* < 0.001; *n* = 25 [5 healthy donors, 5 BC patients per BC stage (I-IV)].

We determined the exosome concentration by NTA, and we found that, compared with healthy donors, the quantity of exosomes found in the serum of BC patients in stages I (*P* < 0.05), III (*P* < 0.001), and IV (*P* < 0.001) was significantly increased (Figure 3), suggesting that breast cancer increases exosome release in BC patients.

### 3.4. Quality of Small RNA Extracted from Exosomes

**Figure 4:**
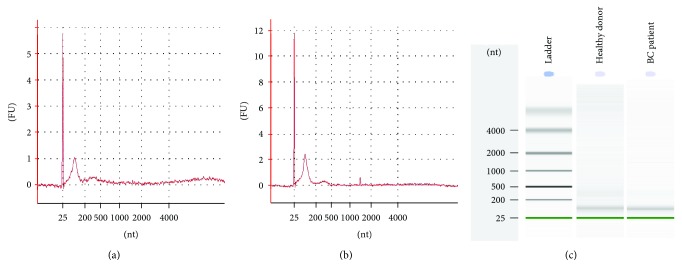
Quality of small RNA isolated from exosomes. Representative electropherogram of RNA quality extracted from exosomes derived of (a) healthy donors and (b) BC patients. All samples were analyzed by the Agilent 2100 Bioanalyzer (Agilent Technologies, Santa Clara, CA) with the RNA 6000 Pico Kit (Agilent Technologies). The *Y*-axis represents fluorescence units (FU) and the *X*-axis shows nucleotides (nt). The peak observed between 25 nt and 200 nt correspond to the small RNA region. Peaks in the regions around 1500 nt and 4000 nt relative to 18S and 28S ribosomal RNAs, respectively, were not or barely detected. The peak of 25 nt refers to the marker. (c) Virtual gel of the data is shown in the electropherograms.

After exosome characterization, RNA was extracted from exosomes and the analysis of RNA quality was done as described in Materials and Methods. We observed a peak between 25 and 200 nt that corresponds to the small RNA region. Peaks in the regions of 18S and 28S ribosomal RNAs were small or not detected in healthy donors (Figure 4(a)) and BC patient samples (Figure 4(b)). Our results indicate that we isolated specifically small RNAs from exosomes.

### 3.5. miR-145, miR-155, and miR-382 in Exosomes of BC Patients

**Figure 5:**
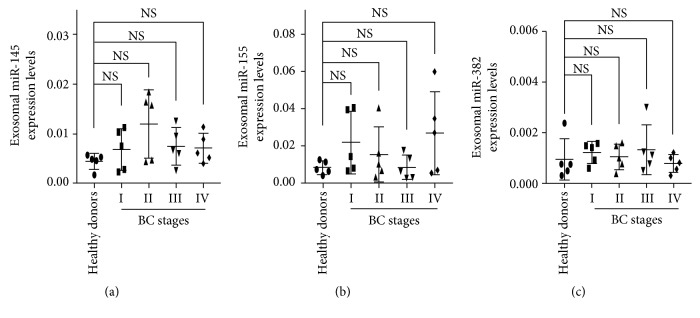
miR-145, miR-155, and miR-382 in exosomes. Expression levels of serum exosomal miR-145 (a), miR-155 (b), and miR-382 (c) in healthy donors and BC patients were determined by qRT-PCR. miR-16 was used as an endogenous control. All qRT-PCR reactions were performed in triplicate, and values are presented as mean ± SD. Since the data were non normally distributed, Kruskal-Wallis followed by Mann-Whitney test was performed; *n* = 25 [5 healthy donors, 5 BC patients per BC stage (I-IV)].

We performed qRT-PCR to assess the presence of miR-145, miR-155, and miR-382 in the exosomes isolated from serum of BC patients and healthy donors. We detected miR-145 (Figure 5(a)), miR-155 (Figure 5(b)), and miR-382 (Figure 5(c)) in exosomes from BC patients and healthy donors. The expression levels of miR-145, miR-155, and miR-382 were normalized using miR-16 because our CT values show that this miRNA is stably expressed in all of our samples (Supplementary Table 1 and Supplementary Figure 1). Nevertheless, compared with healthy donors, the relative expression levels of these three miRNAs in BC patients in stages I-IV were not significant (*P* > 0.05 for each miRNA and stage). Although we did not find a differential expression for these exosome serum miRNAs in BC patients compared to healthy donors, our results indicate that miR-145, miR-155, and miR-382, previously proposed as potential breast cancer biomarkers [20], are present in the exosomes of BC patients but not in a selective manner compared to healthy donors.

## 4. Discussion

Our study showed that serum-circulating miR-145, miR-155, and miR-382 are present in exosomes. These miRNAs were previously proposed as noninvasive biomarkers to distinguish between BC patients and healthy individuals. We also found that exosome concentration was significantly increased in BC patients.

For the isolation of exosomes, the ultracentrifugation-based method is commonly used. An alternative is the commercially available kit ExoQuick based on the precipitation of exosomes, which has been successfully used to isolate exosomes [12, 13, 19]. Using this method, we successfully extracted exosomes from the serum of BC patients and healthy donors.

The storage time and temperature of the biofluid from which exosomes are isolated can affect their stability. We used the recommended storage temperature for exosomes, -80°C [19, 29]. Under these conditions, exosomes are stable for three months [30]; we stored the serum samples for 2 months, to ensure that the stability of exosomes was not compromised.

Exosomes are extracellular vesicles that share some characteristics like round shape, a maximum size of 150 nm, and the presence of proteins such as CD9, CD63, CD81, CD82, Alix, Tsg, and Hsp70 that have been commonly used as exosome markers [13, 15, 31]. Our data are consistent with these reports, since we observed the characteristic round shape and size for the exosomes isolated from the serum of BC patients (119.84 nm) and healthy donors (115.4 nm) by NTA. The size of BC exosomes by AFM (134.4 nm) was similar to the size found by NTA and within the range of the size described [13–15]. We detected vesicles that were 200 nm or larger, which could be caused by aggregation of exosomes, as observed during AFM analysis. Furthermore, we detected the expression of the exosome markers CD63, Alix, Tsg, CD9, and CD81 in all of our exosome samples. Our findings support that the vesicles we isolated from the serum of BC patients and healthy donors correspond to exosomes.

Exosomes can be released by normal cells as well as cancer cells; however, it has been shown in ovarian, lung, colorectal, and breast cancer that cancer cells secrete a higher quantity of exosomes compared with normal cells [32, 33]. Similarly, we found a significant increase in exosome concentration in BC patient samples versus healthy donors, which support the association between exosome concentration and BC. Therefore, more research is needed to elucidate the different miRNAs packaged in the secreted exosomes of BC patients and healthy individuals and how this content may play a role in BC progression.

Initially, exosomes were considered garbage bags that discard excess or nonfunctional molecules (DNA, RNA, or proteins) from cells, but recently, they have emerged as mediators in cell-to-cell communication [15, 34]. Moreover, it has been shown that exosomes can load and transport different molecules like DNA, proteins, lipids, mRNAs, and miRNAs [12, 13]. miRNAs are one of the most attractive cargos of exosomes because they can present different expression patterns under different health conditions, and so they have gained importance as noninvasive biomarkers for diseases such as cancer. In the present study, we extracted small RNA from exosomes, using a method based in column (miRCURY™), which has been reported to have the highest RNA yield in exosomes compared with TRIzol®, TRIzol® + cleanup miRNeasy, mirVana™, RNeasy, and modified RNeasy [35]. Additionally, miRCURY™ together with TRIzol® presented the highest small RNA yield, resulting in exosomes with little or no ribosomal RNA [35]. Likewise, we observed in all of our samples good quality with a peak in the small RNA region and barely or no ribosomal RNA.

The normalization strategy represents a technical challenge in miRNA expression studies, because there is no consensus on a reliable endogenous or exogenous reference gene [36]. miR-16 shows a stable expression and is recommended as a suitable endogenous reference gene for normalization in different cancer studies [19, 37, 38]. In agreement with these reports, we found that miR-16 was stably expressed in our samples, because the CT values obtained from BC patients (stages I, II, III, and IV) were not significantly different (*P* > 0.05) in comparison to the control group (Supplementary Table 1 and Supplementary Figure 1).

miRNAs can be found in different biofluids like urine, plasma, and serum, in which they exhibit high stability because they can be transported and protected from RNase activity through exosome packaging, lipoprotein binding, or association with Argonaute2 (Ago2) [4, 11]. Here, we detected the presence of serum-circulating miR-145, miR-155, and miR-382, in all of the exosome samples, indicating that these miRNAs are packaged in exosomes. Our previous study showed an overexpression of miR-145, 155, and 382 in serum of BC patients [20]. However, in the present study we could not find a significant difference in the expression of these miRNAs packaged into the exosomes of BC patients compared with healthy donors. A previous publication showed that the majority of miRNAs are transported by exosomes in serum and saliva [39]. In contrast, there are some reports that suggest that most of the circulating miRNAs are predominantly associated with Ago-2 and a smaller number of specific miRNAs are packaged by exosomes [40, 41]. Our results could be explained in the sense that not all miRNAs are selectively packaged in exosomes and probably miR-145, miR-155, and miR-382 are being preferentially associated to Ago2 in serum of BC patients. Therefore, further research is needed to elucidate whether a differential expression of these miRNAs is found in association with Ago2 in the serum of BC patients compared to healthy donors. The mechanisms of the selective packaging of miRNAs remain unknown to date [10, 34].

A large number of serum-circulating miRNAs are deregulated in BC compared with healthy controls and identified as biomarkers in BC diagnosis and prognosis [11, 16, 18, 42]. This study is one of the few reports that analyze expression levels of miRNAs in exosomes isolated from serum of BC patients. In one of the reports, the expression levels of four exosomal miRNAs (miR-340-5p, miR-17-5p, miR-130a-3p, and miR-93-5p) were found significantly associated with BC recurrence [19]. miR-182 was found in exosomes of human serum and in breast cancer cells [43]. Another report showed that the expression level of serum exosomal miR-373 was significantly higher in triple negative compared with luminal BC carcinoma [44]. Although we could not find a differential expression of miR-145, miR-155, and miR-382 in BC patients versus healthy donors, our findings contribute to the expansion of our knowledge regarding to the expression of the serum-exosomal miRNAs in BC patients because, to the best of our knowledge, this is the first study that reports the presence of miR-145, miR-155, and miR-382 in exosomes isolated from serum of BC patients.

## 5. Conclusion

Our results show that miR-145, miR-155, and miR-382 are packaged and transported to the serum by exosomes, but not in a preferential manner in BC patients compared to healthy donors. Moreover, our data supports the association between exosome concentration and BC, opening the possibility to study how miRNAs packaged into exosomes play a role in BC progression.

## Figures and Tables

**Table 1:** Clinical characteristics of BC patients and healthy donors.

	Healthy donor (*N* = 5)	Stage I (*N* = 5)	Stage II (*N* = 5)	Stage III (*N* = 5)	Stage IV (*N* = 5)
*Age (years)*					
Mean	52	68	42	47	60
*Treatment*					
Yes		2	3	3	5
No		3	2	2	
*Comorbidities*					
Diabetes	1			1	
Allergies	1				
Hypercholesterolemia	1				
Hypertension		1		1	
Ankylosing spondylitis				1	
Osteoporosis		1			
Hypothyroidism					1

## Data Availability

The data used to support the findings of this study are available from the corresponding author upon request.

## Supplementary Materials

Supplementary Materials Supplementary 1. Table 1: CT values of miR-16 from BC patients (stages I, II, III, and IV) and healthy donors. Supplementary 2. Figure 1: expression of miR-16 in BC patients (stages I, II, III, and IV) and healthy donors. CT values are presented as mean ± SD. Statistical analysis was conducted by the Student *t*-test, *P* > 0.05; *n* = 25 [5 healthy donors, 5 BC patients per BC stage (I-IV)].
