# Supplementary material for: Presence of Circulating miR-145, miR-155, and miR-382 in Exosomes Isolated from Serum of Breast Cancer Patients and Healthy Donors
Source: Dis Markers. 2019 Feb 12;2019:6852917. doi: 10.1155/2019/6852917 (PMC6390256; doi:10.1155/2019/6852917)
Supplement: Supplementary Materials — Supplementary 1. Table 1: CT values of miR-16 from BC patients (stages I, II, III, and IV) and healthy donors. Supplementary 2. Figure 1: expression of miR-16 in BC patients (stages I, II, III, and IV) and healthy donors. CT values are presented as mean ± SD. Statistical analysis was conducted by the Student t-test, P > 0.05; n = 25 [5 healthy donors, 5 BC patients per BC stage (I-IV)]. [file 6852917.f1.docx]

**Supplementary Table 1: CT Values of miR-16 from BC patients (stages I, II, III, and IV) and Healthy donors**

|  | Patient number | CT Values of miR-16 | Average of CT Values |
| --- | --- | --- | --- |
|  |  |  |  |
| Healthy  donors | 1 | 26.08 | 25.99 |
|  | 1 | 25.80 |  |
|  | 1 | 26.09 |  |
|  | 2 | 21.20 | 21.25 |
|  | 2 | 21.36 |  |
|  | 2 | 21.19 |  |
|  | 3 | 21.39 | 21.55 |
|  | 3 | 21.57 |  |
|  | 3 | 21.67 |  |
|  | 4 | 22.76 | 22.93 |
|  | 4 | 23.15 |  |
|  | 4 | 22.86 |  |
|  | 5 | 21.88 | 21.91 |
|  | 5 | 21.97 |  |
|  | 5 | 21.88 |  |
| Stage I | 1 | 23.22 | 23.22 |
|  | 1 | 23.18 |  |
|  | 1 | 23.24 |  |
|  | 2 | 23.20 | 23.29 |
|  | 2 | 23.37 |  |
|  | 2 | 23.31 |  |
|  | 3 | 21.48 | 21.63 |
|  | 3 | 21.74 |  |
|  | 3 | 21.67 |  |
|  | 4 | 23.94 | 24.06 |
|  | 4 | 24.13 |  |
|  | 4 | 24.12 |  |
|  | 5 | 21.18 | 21.16 |
|  | 5 | 21.12 |  |
|  | 5 | 21.17 |  |
| Stage II | 1 | 23.90 | 23.90 |
|  | 1 | 23.79 |  |
|  | 1 | 24.00 |  |
|  | 2 | 23.66 | 23.47 |
|  | 2 | 23.41 |  |
|  | 2 | 23.35 |  |
|  | 3 | 21.07 | 21.15 |
|  | 3 | 21.13 |  |
|  | 3 | 21.24 |  |
|  | 4 | 21.57 | 21.69 |
|  | 4 | 21.71 |  |
|  | 4 | 21.77 |  |
|  | 5 | 19.63 | 19.75 |
|  | 5 | 19.76 |  |
|  | 5 | 19.87 |  |
| Stage III | 1 | 20.52 | 20.62 |
|  | 1 | 20.67 |  |
|  | 1 | 20.67 |  |
|  | 2 | 22.54 | 22.69 |
|  | 2 | 22.81 |  |
|  | 2 | 22.72 |  |
|  | 3 | 22.78 | 22.82 |
|  | 3 | 22.68 |  |
|  | 3 | 23.01 |  |
|  | 4 | 21.07 | 21.07 |
|  | 4 | 20.97 |  |
|  | 4 | 21.17 |  |
|  | 5 | 22.22 | 22.16 |
|  | 5 | 22.10 |  |
|  | 5 | 22.15 |  |
| Stage IV | 1 | 22.92 | 22.94 |
|  | 1 | 22.87 |  |
|  | 1 | 23.04 |  |
|  | 2 | 25.04 | 24.91 |
|  | 2 | 24.90 |  |
|  | 2 | 24.80 |  |
|  | 3 | 23.34 | 23.35 |
|  | 3 | 23.40 |  |
|  | 3 | 23.31 |  |
|  | 4 | 22.36 | 22.27 |
|  | 4 | 22.12 |  |
|  | 4 | 22.32 |  |
|  | 5 | 21.56 | 21.60 |
|  | 5 | 21.72 |  |
|  | 5 | 21.52 |  |

Supplementary Figure 1: Expression of miR-16 in BC patients (stages I, II, III, and IV) and healthy donors. CT values are presented as mean ± SD. Statistical analysis was conducted by Student *t*-test, P > 0.05; *n=*25 [5 healthy donors, 5 BC patients per BC stage (I-IV)].
